# Pre-analytical considerations for microRNA quantification in childhood leukemia research

**DOI:** 10.14440/jbm.2025.0001

**Published:** 2025-05-27

**Authors:** Ioannis Kyriakidis, Iordanis Pelagiadis, Eftichia Stiakaki

**Affiliations:** Department of Pediatric Hematology-Oncology and Autologous Hematopoietic Stem Cell Transplantation Unit, University Hospital of Heraklion and Laboratory of Blood Diseases and Childhood Cancer Biology, School of Medicine, University of Crete, Heraklion 71003, Greece

**Keywords:** MicroRNAs, Pre-analytical phase, Biomarker, Leukemia, Childhood, Adolescence, Exosomes, Confounding factors

## Abstract

**Background:**

MicroRNAs (miRNAs) have gained significant attention as potential biomarkers in childhood leukemia, offering insights into diagnosis, prognosis, and therapeutic targeting. However, the clinical translation of miRNA biomarkers faces several challenges, including inconsistencies in results caused by methodological differences, sample processing variability, and a lack of standardized normalization techniques. In addition, miRNA profiles are highly cell-specific, with unique signatures for different blood cell types and leukemic blasts, reflecting their distinct biological roles and disease states. Pre-analytical factors are critical in ensuring the accuracy and reproducibility of miRNA quantification. The selectively enriched and highly stable exosomal miRNAs have shown great promise for studying intercellular communication and disease-specific miRNAs. The selection of the analytical matrix should align with the specific objectives of the research or diagnostic application. Addressing technical challenges and recording potential confounding variables (*e.g*., age, gender, ethnicity, body mass index, menstrual cycle, fasting, circadian rhythm, comorbidities, medications, smoking, and physical activity) are essential to enhancing the reproducibility and reliability of miRNA biomarkers.

**Objective:**

This review aims to highlight the challenges facing miRNA quantification in childhood leukemias, scrutinizing all relevant pre-analytical issues, including the preferred specimen source, leukemic blast type and count, the selection of suitable blood components, the impact of time and freeze–thaw cycles, collection, processing, and storage variables.

**Conclusion:**

Further research is needed to standardize methodologies and expand our understanding of miRNA interaction networks, ultimately advancing the application of miRNAs in childhood leukemia research.

## 1. Introduction

The discovery of microRNAs (miRNAs) has revolutionized our knowledge of gene regulation and led to Victor Ambros and Gary Ruvkun being awarded the Nobel Prize in Physiology or Medicine in 2024. Over the past three decades, it has become evident that genes are not merely passive entities residing in the cell nucleus that transcribe messenger RNA (mRNA) to initiate the production of their corresponding proteins.^1^ MiRNAs are short, non-coding RNA molecules, measuring approximately 22 nucleotides in length, and play a pivotal role in the regulation of gene expression. They function by either promoting the degradation of target mRNAs or inhibiting their translation.

Chromatin reorganization followed by gene silencing is another feature of miRNA-mediated mechanisms.^2^ In normal hematopoiesis, miRNAs are essential for maintaining the balance between self-renewal and differentiation of hematopoietic stem cells, ensuring the proper development of blood cell lineages. Specific miRNAs exhibit dynamic expression gradients along hematopoietic differentiation trajectories to regulate critical genes involved in the proliferation, differentiation, and apoptosis of hematopoietic cells, thereby orchestrating the orderly production of platelets (PLT), red blood cells (RBC), and white blood cells (WBC).^3^,^4^

In childhood leukemias, miRNAs have been found to play significant roles in the pathogenesis, diagnosis, and progression of the disease. Aberrant expression of specific miRNAs can disrupt normal hemopoietic processes, leading to leukemogenesis. For example, miRNAs can act as oncogenes or tumor suppressors, and their dysregulation can contribute to the uncontrolled proliferation and survival of leukemic cells.^5^-^7^ Polymorphisms in miRNA genes and miRNA-processing genes have also been associated with susceptibility to childhood acute lymphoblastic leukemia (ALL).^7^,^8^ In diagnosis, specific miRNA expression profiles can serve as biomarkers for acute leukemias, helping to distinguish between different subtypes and predict patient outcomes.^7^ Moreover, miRNA signatures have been linked to relapse, as changes in their expression can reflect the presence of measurable residual disease or the emergence of treatment-resistant leukemic clones.^6^

miRNAs are being explored as potential therapeutic targets and prognostic tools in childhood ALL, offering insights into drug resistance mechanisms and novel treatment approaches. Their study holds promise for enhancing diagnosis, treatment, and survival outcomes, making them key players in personalized medicine for ALL.^9^,^10^ Many registered clinical trials are currently conducting miRNA research in various settings (*e.g*., NCT03000335, NCT06649253, NCT00526084, NCT00896766, and NCT01282593 in ALL; NCT01298414 and NCT00900224 in acute myeloid leukemia [AML]; NCT01229124 in infantile AML; NCT01541800 in leukemia and lymphoblastic lymphoma; NCT05477667 in acute leukemia and non-Hodgkin lymphoma; NCT05809050 and NCT02613962 in acute leukemias; NCT01511575 in Down syndrome with AML or transient myeloproliferative disorder; https://clinicaltrials.gov/, last accessed on December 30, 2024). The aforementioned clinical trials involve bone marrow (BM) and peripheral blood samples and investigate diagnostic, relapsed, and remission samples. To date, miRNA signatures have been utilized for the diagnosis, high-risk stratification, and prognosis of hematological malignancies in childhood and adolescence, along with their association with treatment response, toxicity, and late effects.^7^,^11^-^14^ Several parameters play a crucial role in miRNA quantification. Therefore, research on miRNAs should have a precise goal setting for an optimal design of the corresponding experiments.

**Figure 1:**
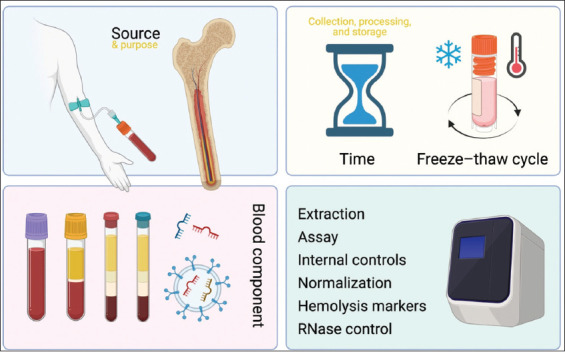
Pre-analytical and analytical factors for investigating miRNA levels in childhood leukemias. Created in BioRender. Kyriakidis, I. (2025) https://BioRender.com/4xf9qnp.

This review highlights the challenges confronting miRNA quantification in childhood leukemias (Figure 1). All relevant pre-analytical factors are being addressed, including age, gender, preferred source of the specimens, selection of the suitable blood component, the impact of time and freeze–thaw cycles, collection, processing, and storage variables.

miRNA research requires a careful protocol design, depending on the aims of each study. The highlighted pre-analytical factors influence the accuracy and reproducibility of miRNA quantification in childhood leukemia research, including the selection of appropriate biological materials, the role of sample storage and processing conditions, the impact of time and freeze–thaw cycles, and the importance of avoiding contamination from RBCs and PLTs.

## 2. miRNAs levels in blood components

Besides the intracellular miRNA content, miRNAs circulate in a highly stable, cell-free form in the blood and thus can be detected in plasma and serum.^15^ MiRNAs can be measured in whole blood and all blood components. Plasma is the cell-free supernatant obtained after centrifuging blood collected in an anticoagulant-containing tube. The serum refers to the cell-free supernatant acquired after centrifuging blood left to clot spontaneously in a tube with no anticoagulant. Cells display significantly different concentrations of most miRNAs than serum or plasma, and cellular carryover is a potential confounding factor associated with the risk of contaminating the plasma supernatant by cells from the cellular pellet during aspiration.^16^ Likewise, clot formation in serum samples can result in cell lysis and enrichment of miRNAs originating from RBCs and PLTs.^17^

Table 1 presents a comprehensive listing of the concentrations of selected miRNAs in healthy and leukemia tissue: miR-155-5p is a leukemia-specific miRNA,^18^ miR-451a is specific for RBCs and thus indicative of hemolysis,^19^ miR-126 is specific for PLTs (but also involved in hematopoietic stem cell quiescence and B-ALL pathogenesis),^20^ and miR-16 and miR-191 have been used as references in many studies.^21^,^22^ Expression values have been obtained from the miRNA tissue expression database (https://dianalab.e-ce.uth.gr/mited/#/; last accessed on December 30, 2024),^23^ and they are presented in reads per million. Whole blood, serum, and plasma values correspond to peripheral blood. BM values correspond to whole blood drawn with BM aspiration, and mononuclear cells (MNCs) isolated from the peripheral blood are cited as PBMCs. Table 1 also reports the expression values of the selected miRNAs in several cell lines: Jurkat, HPB-ALL, MOLT-4, and CCRF-CEM cell lines corresponding to T-ALL, and the THP-1 cell line derived from an acute monocytic leukemia patient.

**Table 1:** Expression levels of selected microRNAs in various tissues and cell lines

MicroRNA	Expression in healthy tissue (RPM)		Expression in leukemia cell lines (RPM)	
miR-155-5p	Whole blood	18.745	T-ALL blood	3,312.997
	Bone marrow	300.890	AML blood	57.445
	PBMC	2,572.866	Jurkat	202.452
	WBC	964.470	HPB-ALL	5,288.282
	RBC	13.650	MOLT-4	2,646.560
	Serum	51.847	CCRF-CEM	3,757.320
	Plasma	87.531	THP-1	3,864.720
miR-451a	Whole blood	87,853.366	T-ALL blood	1,592.883
	Bone marrow	1.030	AML blood	34,131.482
	PBMC	3,527.091	Jurkat	1.250
	WBC	9,102.940	HPB-ALL	0.483
	RBC	662,939.000	MOLT-4	0.120
	Serum	280,012.557	CCRF-CEM	0.120
	Plasma	94,051.987	THP-1	7.802
miR-126-3p	Whole blood	237.930	T-ALL blood	132.865
	Bone marrow	2.720	AML blood	2.295
	PBMC	2,818.020	Jurkat	38.088
	WBC	2,926.840	HPB-ALL	0.160
	RBC	1,867.073	MOLT-4	48.150
	Serum	5,576.083	CCRF-CEM	2,543.070
	Plasma	5,856.687	THP-1	151.920
miR-16-5p	Whole blood	2,800.655	T-ALL blood	39,719.386
	Bone marrow	2,689.910	AML blood	1,847.871
	PBMC	30,479.586	Jurkat	34,157.702
	WBC	45,330.320	HPB-ALL	58,397.158
	RBC	87,337.103	MOLT-4	2,175.480
	Serum	23,476.967	CCRF-CEM	5,001.020
	Plasma	22,073.606	THP-1	35,461.452
miR-191-5p	Whole blood	12,416.874	T-ALL blood	39,395.690
	Bone marrow	53,160.650	AML blood	17,212.518
	PBMC	75,160.631	Jurkat	43,368.960
	WBC	106,072.940	HPB-ALL	37,202.795
	RBC	13,132.583	MOLT-4	2,283.050
	Serum	4,438.683	CCRF-CEM	7,885.540
	Plasma	8,935.388	THP-1	219,099.452

Abbreviations: ALL: Acute lymphoblastic leukemia; AML: Acute myeloid leukemia; PBMC: Peripheral blood mononuclear cells; RBC: Red blood cell; RPM: Reads per million; WBC: White blood cell.

In general, serum and plasma miRNA levels are considered to be equivalent, but PLTs contain a broad spectrum of miRNAs, and these may be released into the serum during coagulation, together with miRNAs from RBCs and WBCs.^24^ There is mounting literature comparing plasma and serum samples side-by-side, which concluded in little or no difference in extracellular miRNA quantification. However, higher concentrations were consistently found in sera.^25^ The biofluid type seems to significantly impact identified miRNAs, because substantial differences in miRNA expression profiles can be noted even when plasma and sera were collected from the same patients and compared with the same technical approach.^17^,^26^ More miRNAs are expected to be detected in plasma than in sera, while miR-451a, miR-16-5p, miR-223-3p, and miR-25-3p are highly abundant and differentially expressed between the two biofluids.^17^ Higher levels of PLT-derived miRNAs (miR-185-5p, miR-22-3p, and miR-320b) are anticipated in plasma than in serum, while levels of RBC-derived miRNAs (miR-451a, miR-486-5p, and miR-92a-3p) are not expected to differ significantly in serum and plasma.^27^

In the context of translational studies, plasma should be preferred over serum for miRNA investigations.^28^ The difference between serum and plasma miRNA concentration showed some associations with miRNA from PLTs, indicating that the coagulation process may affect the spectrum of extracellular miRNAs in blood.^29^ PLT and its microvesicles constitute a significant source of miRNAs, and six among 675 PLT-derived miRNAs seem to reflect PLT condition and function: let-7b-5p, miR-16-5p, miR-17-5p, miR-107, miR-126-3p, and miR-191-5p.^30^

The type of anticoagulant used in plasma collection tubes is also an important factor. While ethylenediaminetetraacetic acid (EDTA) and citrate are acceptable anticoagulants for downstream quantitative reverse transcription-polymerase chain reaction (qRT-PCR), heparin potently inhibits subsequent PCR processes. Regarding serum collection, it is crucial to standardize the type of collection tube used (with or without a gel separator) to avoid confounding the results.^16^ Novel high-throughput microfluidic extraction methods of PLT-free plasma for miRNA and extracellular vesicle analysis provide a convenient and automated centrifugation-free workflow for point-of-care diagnostics.^31^ Moreover, results for specific miRNAs warrant interpretation with caution. For example, ALL cells display a significantly higher cell-to-plasma ratio of miR-92a, indicating that leukemic cells retain this miRNA.^32^

The ratio of miR-451 to miR-23a-3p assessed by PCR analysis has been widely used as a quality measure, and a ratio of >7 has been used as a marker of hemolysis.^33^ Unfortunately, this metric is not directly applicable in Nanostring analyses because PCR-measured miRNA expression depends on amplification steps and the non-linear relationship between the Ct value and the transcript number.^17^ The measurement of plasma absorbance at 414 nm (the hemoglobin absorbance peak, should be <0.3 absorbance unit and ideally <0.072) is also a prerequisite.^34^

Extracellular miRNA content displays relative stability mainly attributed to the protection from RNases following their packaging in membrane-coated (*e.g*., apoptotic bodies 0.1 – 5 μm, microvesicles >0.5 μm, ectosomes 100 – 600 nm, oncosomes 1 – 10 μm, exosomes 30 – 150 nm, prostasomes 50 – 500 nm, melanosomes >0.5 μm, and “PLT dust” microvesicles ~130 – 500 nm) or membrane-free particles (*e.g*., high- and low-density lipoproteins, RNA-binding proteins, such as argonautes 1 [AGO1] and AGO2, nucleophosmin 1, Tamm–Horsfall protein, and exomeres of ~35 nm).^35^ Cell-free miRNAs can also be detected in circulating exosomes associated with the surface of blood cells.^36^ Interestingly, exosomal miRNAs exhibit extra stability under different storage conditions.^37^ With advancements in exosome biology, exosome-based therapies and diagnostics could become integral to leukemia management, offering more effective and personalized strategies to address this complex disease.^38^

**Figure 2:**
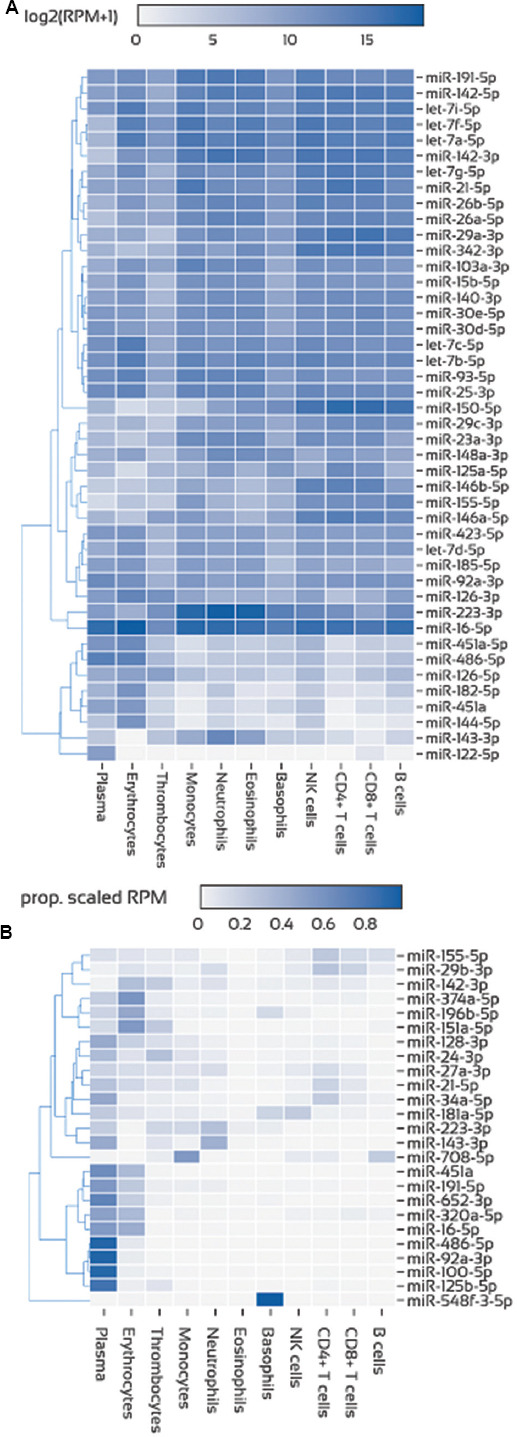
Variations in microRNA levels across blood-derived fractions. (A) Expression of the union of the most abundant microRNAs per blood component. (B) Relative contribution (scaled proportion) of blood components to expression in whole blood. Created in BioRender. Kyriakidis, I. (2025) https://BioRender.com/70lk04m. Abbreviation: RPM: Reads per million.

**Figure 3:**
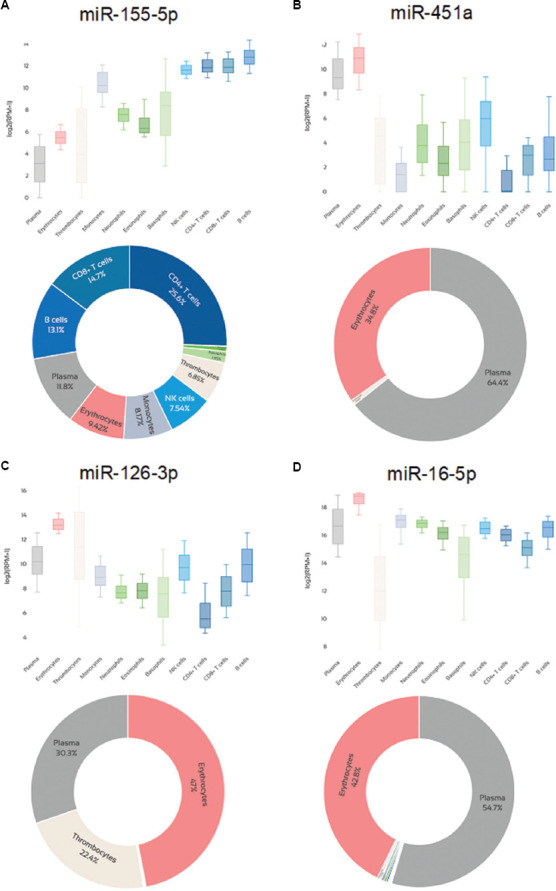
Expression of specific microRNAs (miRNAs) in blood components and relative contribution of blood components to the expression in whole blood for miR-155-5p (A), miR-451a (B), miR-126-3p (C), and miR-16-5p (D). Created in BioRender. Kyriakidis, I. (2025) https://BioRender.com/0iqx6i0.

Figure 2 illustrates the levels of the most common miRNAs in each blood component and the relative contribution of blood components to the expression of selected miRNAs that seem to play significant roles in childhood leukemias. Figure 3 demonstrates the expression of leukemia-specific miR-155-5p, RBC-specific miR-451a, PLT-specific miR-126-3p, and miR-16-5p in several blood components and the relative contribution of each blood component to their corresponding total expression in the whole blood. Data were extracted from miR-Blood (https://mir-blood.com/; last accessed on December 30, 2024), a high-quality miRNA expression atlas for the major components of human peripheral blood: Plasma, erythrocytes, thrombocytes, monocytes, neutrophils, eosinophils, basophils, natural killer cells, B-cells, cluster of differentiation (CD)4^+^ and CD8^+^ T-cells.^39^

The selection of miRNAs (Figure 2B), which play significant roles in childhood leukemias, was based on the literature, the confirmation of these miRNA signatures in large RNA-seq datasets (unpublished data), and the miRNA panel developed by our laboratory for the “microRNA levels in childhood ALL and survival (MICRO-CALLS)” project. Key miRNAs in childhood ALL include miR-21-5p, miR-24-3p, miR-100-5p, miR-125b-5p, miR-128-3p, miR-143-3p, miR-155-5p, miR-181a-5p, and miR-708-5p, which are deregulated at diagnosis and associated with disease prognosis. MiRNAs, such as miR-27a-3p, miR-29b-3p, miR-34a-5p, miR-92a-3p, miR-142-3p, miR-151a-5p, miR-196b-5p, miR-223-3p, miR-320a-5p, miR-374a-5p, miR-548f, and miR-652-3p, are linked to disease progression and relapse, acting as valuable prognostic indicators.^6^,^7^ Furthermore, miR-451a and miR-486-5p are recognized as RBC-specific miRNAs, while miR-191-5p and miR-16-5p are frequently used as reference miRNAs in quantitative PCR studies.

## 3. miRNAs levels in BM and peripheral blood

Research on miRNA concentration in childhood leukemias has yielded contradictory results between BM and peripheral blood specimens. A study on 25 children with ALL from Hungary showed that the miRNA expression profile was not significantly different between MNCs of the peripheral blood and the BM from the same patient.^40^ On the contrary, another study from Hungary on 20 children with ALL found no significant correlation between miRNA expressions in platelet-free plasma derived from the BM and the peripheral blood.^41^ In the same context, a study from Turkey on 43 *de novo* childhood ALL cases (utilizing total RNA) reported significant fluctuations between BM and peripheral blood miRNA expressions.^42^ A recent study on 48 childhood ALL cases from India reported significant deviations in miRNA levels between BM and peripheral blood RBC-free samples.^43^

Despite the contradictory findings between BM and peripheral blood miRNA expression profiles, many studies in the literature have included both kinds of samples in the same study.^9^,^44^,^45^ A recent study on 11 children with ALL from Brazil revealed a consistent expression of 12 out of 14 miRNAs in matched BM–peripheral blood MNC samples. Notably, tumor suppressors miR-152-3p and miR-27a-3p had significantly lower expression in BM samples.^46^ Both miRNAs have been previously found to be implicated in the pathogenesis of acute and chronic leukemias.^47^-^49^ In addition, four more leukemia-related miRNAs displayed marginal correlations (i.e., miR-128-3p, miR-181b-5p, miR-363-3p, and miR-708-5p).^46^ BM plasma may show enrichment of certain miRNAs specific to the hematopoietic niche or involved in BM-specific pathologies. Studies on adults have acknowledged the role of the extracellular BM microenvironment and denoted its differences in miRNA levels with blood drawn peripherally.^50^ Table 1 demonstrates the differences in the levels of selected miRNAs measured in the BM and the peripheral blood. Studies utilizing matched BM and peripheral blood MNC samples are not expected to display significantly different miRNA levels unless both conditions described below are unmet.

Disruptions in miRNA biogenesis and post-transcriptional gene regulation can significantly impact hematopoietic differentiation and proliferation, potentially resulting in malignant hematopoiesis. Vice versa, the genomic profile of hematological malignancies can regulate miRNA levels.^51^ It is well-established that every blood cell type exhibits a distinct miRNA signature. Several studies have demonstrated the role of specific miRNAs in hematopoiesis.^3^,^4^ A more diverse range of miRNAs has been identified in monocytes, peripheral macrophages, and common myeloid progenitor cells, suggesting significant implications for research on hematological malignancies originating from these types of cells.^52^ In addition, the expression of miRNAs may be dependent on blast count.^40^ Altogether, the blast type, count, and percentage must be accounted for when designing studies. Based on the data mentioned above, adjustment for the blast percentage or setting a relatively high cutoff for blast infiltration is advised, especially regarding the heterogeneity observed with AML.

The tumor microenvironment in the BM also plays a pivotal role in hematological malignancies. Exposure of ALL cells to primary human BM niche cells (including BM stromal cells and primary human osteoblasts) may result in global alterations in miRNA expression profiles. These changes are associated with the BM microenvironmental interactions, modified cell cycle kinetics, and altered responses to several conventional chemotherapeutic agents.^53^ The expression of miRNAs within the BM tumor microenvironment does not necessarily reflect the circulating levels of that same miRNA.^54^ The role of exosomes in the BM microenvironment is an area of active investigation.^55^ Recent discoveries have ascertained the role of miRNA content within extracellular vesicles in hematological malignancies. These vesicles often carry enriched transcripts that are relevant to leukemia prognosis and the regulation of stem cell niche function. In childhood leukemias, research on the BM microenvironment predominantly aims at identifying potential pharmacological targets and understanding the biology of leukemogenesis.^56^,^57^

Another challenge in miRNA profiling when designing studies on childhood leukemia is the risk of overrepresenting specific molecular subtypes. This issue arises because the biological characteristics of leukemic blasts significantly influence miRNA expression patterns. In diagnostic settings, specific miRNA expression profiles can serve as biomarkers for ALL, helping to distinguish between different subtypes (*e.g*., T-cell immunophenotype, *BCR::ABL1*, or *KMT2A*-rearranged ALL).^7^

## 4. Blood collection, processing, and storage

Current protocols for plasma-based miRNA profiling in diagnostic applications recommend a whole blood holding period spanning from immediately after collection to 26 h. A relevant study demonstrated that whole blood holding time was a major source of variation in 53 out of 179 miRNA levels in specimens held at 4°C for 30 min, 2 h, 6 h, or 24 h before plasma isolation processing.^58^ This discrepancy was mainly attributed to hemolysis. A recent study on time-course changes in levels of total miRNAs in serum and plasma revealed no significant time-dependent changes in serum samples. However, following blood collection, substantial changes in the levels of 37% of miRNAs were noticed in plasma samples during the time course (0 – 3 h). In the latter study, 173 miRNAs tended to decrease with time, while plasma levels of six miRNAs (*i.e*., miR-128-2-5p, miR-3619-3p, miR-3620-3p, miR-4649-5p, miR-4758-3p, and miR-6885-5p) tended to increase with time, presumably due to the endocytosis of miRNA-containing exosomes by blood cells.^27^ Hemolysis and blood storage at room temperature, 4°C, and −20°C negatively affect the accuracy in quantifying circulating miR-451a and miR-423-5p, while miR-199a-3p levels in PLT-free plasma seem to remain stable for at least 72 h at room temperature.^59^ All relevant reports stress the issue of the early preparation of plasma samples after blood collection.

Adherence to the standard operating procedures for miRNA sequencing is critical for minimizing variability and ensuring reproducibility. Key pre-analytical steps include the removal of contaminant cells and PLTs, aliquoting of samples, and storage at −80°C. In addition, plasma collected into EDTA tubes is generally preferred over serum, due to lower background miRNA contamination, and strict control over centrifugation parameters (*e.g*., timing and force) is essential to ensure consistent sample quality. Another key element is the preparation of fresh and sterile reagents the day before sequencing. Reagents such as 1 N NaOH, 400 mM Tris-HCl (pH 8.0), and 10 mM Tris-HCl (pH 8.5) should be freshly prepared using nuclease-free water to prevent nucleic acid degradation.^34^ These solutions must be autoclaved the day before sequencing.

Modern protocols for miRNA quantification suggest blood collection using an appropriately sized needle (*e.g*., as with BD Vacutainer^®^ Safety-Lok^™^ sets) to avoid hemolysis and collection in K_3_- or K_2_-EDTA-containing tubes. Filled tubes should be inverted no more than 10 times and then stored upright for up to 2 h at room temperature (15 – 25°C).^34^ Timely centrifugation at 1,800× *g* for 10 min at room temperature is essential to avoid PLT activation and the release of microvesicles containing miRNAs. It is worth noting that PLTs can be activated at low temperatures and release intracellular proteins, enzymes, and miRNA-containing microvesicles, significantly affecting the samples. Alternatively, processing at low temperatures should be performed only after PLT removal. Since a single centrifugation step may not be adequate to reduce PLT counts to below 10 cells/mL, a second centrifugation step (between 1,200 and 2,500× *g* for 15 min at room temperature) or a filtration step may be required.^60^ Multiple plasma aliquots should be prepared to avoid multiple freeze–thaw cycles (affecting miRNA stability and subsequent analyses) and stored at −80°C to preserve RNA integrity, a temperature that guarantees stability for up to 1 year.^61^ According to an early report, up to eight freeze–thaw cycles are acceptable in miRNA quantification.^62^

An appropriate input amount is crucial for miRNA detection, as insufficient or excessive inputs may impact results due to low miRNA concentrations or elevated levels of inhibitors, such as hemoglobin, immunoglobulin G, and lactoferrin. Plasma components, such as polymerase inhibitors, can coprecipitate with miRNAs and interfere with their detection. Phenol/chloroform extraction and silica-based adsorption may help eliminate these inhibitory substances.^63^,^64^

Other parameters that need to be standardized by further studies include the diurnal variation in miRNA levels and the fasting (versus the non-fasting) state at blood collection, because fatty meals and the resulting lipemia could affect miRNA binding and extraction.^16^ Dietary components, such as resveratrol, curcumin, isoflavones, catechins, indoles, and vitamins, can alter circulating miRNA levels.^65^,^66^ A daily variation of miR-181a and miR-16 expression in human leukocytes has been reported, suggesting the involvement of these miRNAs with the circadian rhythms present in blood cells.^67^ The level of circulating miRNAs can also be confounded by factors such as age, gender, ethnicity, body mass index, menstrual cycle phases, comorbidities, medications, smoking, physical activity, and lifestyle, including quality of sleep, and perceived stress.^65^,^68^-^70^ Earlier reports demonstrated that a significant proportion of miRNAs was influenced by age and gender.^71^ Age-related alterations in WBC differentials, particularly shifts in lymphocyte proportions, lead to changes in miRNA expression profiles, as different WBC types exhibit distinct miRNA signatures. These variations reflect biological divergence and altered regulatory potential in critical networks such as differentiation and growth.^71^,^72^

The association of miRNA expression with response to chemotherapy should be interpreted cautiously, as commonly used medicines, such as paracetamol and aspirin, have been shown to significantly alter miRNA profiles in biological fluids.^73^,^74^ Likewise, children and adolescents with leukemia often receive supportive care with RBC and PLT transfusions, which may interfere with the monitoring of miRNA levels.

## 5. miRNAs quantification methods

Besides pre-analytical challenges, a plethora of analytical issues have been addressed in miRNA quantification in childhood leukemia research, including RNA extraction, selection of internal controls and normalization, hemolysis markers, RNase control, cross-contamination, and assay-specific factors. A recent benchmarking study concluded that the most stable references for miRNA normalization in B-cell precursor ALL studies are miR-103a-3p and miR-532-5p, but the debate on this topic is heated.^21^

Multiple normalizers and adherence to protocols of published high-quality works ensure reproducibility. Several studies in the literature compared the efficiency of different techniques of miRNA quantification.^46^,^69^ The optimal method for miRNA quantification in childhood leukemias depends on the study’s objectives, resource availability, sample volumes, and the level of detail required. The gold standard for miRNA quantification is qPCR thanks to its high sensitivity, specificity, accuracy, and cost-effectiveness, particularly suited for targeted leukemia-related miRNA studies. Limited throughput is a significant setback with this method, which is preferred for studies with small sample sizes.^21^,^69^ Microarrays are another option that enables the simultaneous profiling of hundreds to thousands of miRNAs. They are particularly ideal for the screening of differentially expressed miRNAs. However, microarrays cost more and display low sensitivity for low-abundance miRNAs.^42^

Next-generation sequencing allows the discovery of novel and low-abundance miRNAs, isomiRNAs, and sequence modifications. However, the techniques are costly and require significantly higher sample input than conventional qPCR, along with bioinformatic expertise.^11^ Digital PCR is an ultra-sensitive and accurate method, ideal for detecting miRNAs in samples with very low RNA input. Limited multiplexing capacity and high-cost instrumentation are limitations that render this method inferior to qPCR.^75^ Northern blotting is a low-throughput technique that is used for validation purposes but is labor-intensive, requires large amounts of RNA, and is less sensitive than the other methods.^43^,^76^

It is also important to consider the RNA preparation method when discussing confounding factors in miRNA expression measurements. While poly-A capture is efficient for gene expression analysis and detecting structural aberrations by enriching polyadenylated transcripts, it may introduce bias by overlooking non-polyadenylated miRNAs. In contrast, ribosomal RNA depletion offers a more comprehensive representation of the transcriptome, including miRNAs, and is better suited for detecting a wider range of RNA species, mutations, and polymorphisms, thus providing more accurate and unbiased miRNA quantification.^77^

**Figure 4:**
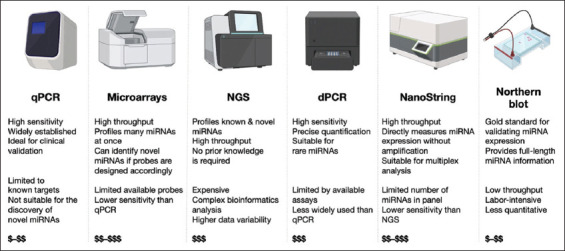
Comparison of available microRNA (miRNA) quantification methods: Approaches, strengths, limitations, and costs. Created in BioRender. Kyriakidis, I. (2025) https://BioRender.com/jfpmb1g. Abbreviations: dPCR: Digital polymerase chain reaction; NGS: Next-generation sequencing; qPCR: Quantitative polymerase chain reaction.

A comparison of the available miRNA expression detection methods, along with their strengths, limitations, and costs, is presented in Figure 4.^76^,^78^,^79^ The analytical and post-analytical factors associated with technical limitations in measuring miRNAs as biomarkers fall beyond the scope of this review and have been extensively reviewed in other literature.^70^

## 6. Conclusion

The significance of miRNAs in disease was recognized with the Nobel Prize in Physiology or Medicine in 2024. With each passing year, we are a step closer to translating miRNA biomarkers from laboratory benches to clinical settings. Numerous reports have failed to validate miRNA biomarkers in childhood leukemias, and technical hurdles must be overcome regarding accuracy and reproducibility. Inconsistent results may be explained by methodological differences, lack of standard methods for normalization, sample processing, and our basic understanding of miRNA interaction networks. It is imperative to record all available variables that could affect outcomes and adjust for the variables that display significant associations. Further in-depth research is needed to account for the potential confounding effects of background factors and to validate the influence of specific test factors or conditions on circulating miRNA levels. Exosome isolation offers the optimal sample for miRNA analysis; however, ongoing advances in the field necessitate continuous review of the emerging literature. The concentration and composition of miRNAs vary between serum and exosomes, reflecting differences in their origin, stability, and packaging mechanisms. Exosomal miRNAs exhibit superior stability and selective enrichment, making them particularly valuable for studying intercellular communication and identifying disease-specific biomarkers. In contrast, serum miRNAs offer a broader but less specific representation of systemic miRNA levels and may be influenced by background noise due to non-specific cellular release. The selection of serum or exosomes as the analytical matrix should align with the particular objectives of the research or diagnostic application. Similarly, the choice of the proper method for miRNA quantification should cater to the needs of the research, whether for discovery, validation, or clinical translation.

To ensure reproducibility in miRNA studies for childhood ALL, selecting an appropriate biological material based on specific research goals is critical. Intracellular miRNA content from leukemic cells, particularly in BM specimens, is essential for pathogenesis studies, while serum and exosomes, especially from peripheral blood, are better suited for clinical applications, including diagnostics and prognostics. Optimal practices involve immediate specimen processing, minimizing confounding factors, adjusting for blast counts, and avoiding hemolysis as well as RBC or PLT contamination. Although analytical and post-analytical challenges exist, they fall beyond the scope of this article; however, they should be addressed in future research to improve miRNA quantification in clinical settings. Future miRNA research in childhood ALL should prioritize standardized exosome protocols and ensure access to affordable miRNA sequencing to facilitate non-invasive and reproducible biomarker discovery, thus enhancing clinical applications for monitoring and personalized treatment.

## Data Availability

Not applicable.
